# Successful Treatment of Concurrently Diagnosed Multiple Myeloma and Myelodysplastic Syndrome With Isolated Del(5q) With Lenalidomide, Bortezomib, and Dexamethasone

**DOI:** 10.7759/cureus.19742

**Published:** 2021-11-19

**Authors:** Nyomi R Washington, Eugen A Shippey, Michael Osswald

**Affiliations:** 1 Internal Medicine, Brooke Army Medical Center, San Antonio, USA; 2 Hematology/Oncology, Brooke Army Medical Center, San Antonio, USA

**Keywords:** del(5q), bortezomib, lenalidomide, myelodysplastic syndrome, multiple myeloma

## Abstract

Lenalidomide is known to be an effective therapy for multiple myeloma and for myelodysplastic syndrome (MDS) with isolated del(5q). We report the case of a patient simultaneously diagnosed with multiple myeloma and myelodysplastic syndrome with isolated del(5q) who was treated successfully with lenalidomide, bortezomib, and dexamethasone. The treatment achieved a stringent complete response of multiple myeloma and a hematologic and cytogenetic response of MDS in three months. Our experience suggests that standard myeloma induction regimens including lenalidomide and a proteasome inhibitor may be considered for treatment of concurrently diagnosed multiple myeloma and MDS with isolated del(5q) and are safe to use in select patients.

## Introduction

There are numerous studies demonstrating the effectiveness of lenalidomide in myelodysplastic syndrome (MDS) with isolated del(5q) and multiple myeloma individually. MDS associated with isolated del(5q) was recognized as a unique entity by the WHO classification of the myeloid neoplasms in 2002 [1]. Typical features of this syndrome include refractory macrocytic anemia without thrombocytopenia, less than 5% blasts in the bone marrow, and an elevated number of hypolobated megakaryocytes. The disease is associated with a fairly indolent course and a better prognosis than other forms of MDS. In 2005, lenalidomide was approved by the FDA to treat patients with MDS with isolated del(5q) based on results of a multicenter, single-arm, open-label study by List et al. in transfusion-dependent patients with the syndrome [2]. With treatment, the patients required fewer blood transfusions and demonstrated reversal of cytogenetic abnormalities.

Lenalidomide is also a well-established therapy for multiple myeloma. It is approved for this indication based on data like that from a phase II trial of lenalidomide in myeloma patients by Richardson et al., showing an 81% objective response rate [3]. It is now regularly incorporated into standard multi-drug induction regimens, used as a single agent in less aggressive treatment regimens, and is an option for maintenance after autologous stem cell transplantation for patients with multiple myeloma.

There have been few reports describing multiple myeloma and MDS with isolated del(5q) co-occurring in the same patient. The first patient to have both conditions concurrently was reported by Rios et al. in 2000 [4]. Since then, two other cases describing concurrently diagnosed multiple myeloma and MDS with isolated del(5q) have been reported in the literature [5,6]. Treatment approaches tried in these three cases include steroids and lenalidomide used at varying doses with varying levels of success. The current case report adds to this small body of literature and demonstrates a different, more intense treatment approach that was used with success. 

An abbreviated version of this case report was previously presented as an abstract in Blood (2020) 136 (Supplement 1): 17-18. Since its presentation, we have updated this report with new data regarding the depth and duration of the patient's response to treatment.

## Case presentation

We report the case of a 74-year-old female who was referred to hematology for worsening chronic macrocytic anemia with a hemoglobin of 9.4 g/dL. A serum protein electrophoresis (SPEP) demonstrated an IgG kappa monoclonal spike of 4.7 g/dL. Free light chain analysis showed a kappa/lambda ratio of 36.7. The patient was mildly hypercalcemic at 10.6 g/dL. She had no renal insufficiency. Platelet and WBC counts were normal. There were no osteolytic lesions on skeletal survey and a whole-body PET scan identified no bony disease or plasmacytomas. β-2 microglobulin level was 3.7 mg/L and albumin was 3.3 g/dL.

Bone marrow biopsy revealed 60% plasma cells in a 70% cellular marrow. Granulocytic and megakaryocytic dysplasia was identified. Fluorescence in situ hybridization returned (FISH) showing a 4:14 translocation in 72% of analyzed nuclei and monosomy 13 in 61% of nuclei analyzed consistent with an unfavorable risk profile for multiple myeloma. Chromosome analysis also revealed a 5q deletion in 15 of 20 analyzed cells. Bone marrow blasts were measured at 1%.

Therefore, the patient concurrently met diagnostic criteria for stage II IgG kappa multiple myeloma per the Revised International Staging System (R-ISS) and low-risk MDS with isolated del(5q) per the 2016 WHO classification of MDS. Her Revised International Prognostic Scoring System Score (IPSS-R) was 2. She was started on lenalidomide 25 mg daily, bortezomib 1.3 mg/m2 on days 1, 4, 8, and 11, and dexamethasone 20 mg on days 1, 8, and 15 of a 21-day cycle.

After four cycles of therapy, serum immunofixation electrophoresis showed an unquantifiably low IgG kappa monoclonal spike and the patient's kappa/gamma light chain ratio had normalized to 1.1 (Table 1). Hemoglobin and calcium returned to normal. On repeat bone marrow biopsy, there was normocellular marrow with 4% polytypic plasma cells. Kappa/lambda immunohistochemistry showed a normal ratio. No dysplasia was identified and bone marrow blasts were 1.5%. FISH and chromosome analysis were normal. Therefore, the patient achieved a stringent complete response to therapy for multiple myeloma according to International Myeloma Working Group criteria within three months. She met International Working Group criteria for hematologic and cytogenetic response of her MDS.

**Table 1:** Key markers showing improvement after four cycles of therapy

	At Diagnosis	After Four Treatment Cycles
Hemoglobin	9.4 g/dL	11.4 g/dL (Normal)
Calcium	10.6 g/dL	9.0 g/dL (Normal)
IgG Kappa M-spike	4.7 g/dL	Unquantifiably low
Light chain ratio	36.7	1.18 (Normal)
Marrow plasma cells	60%	4%, polytypic
Marrow dysplasia	Present	None
FISH	4:14 [72%], -13 [61%]	Normal
Chromosome analysis	del(5q) [15/20]	Normal

After eight cycles of induction, the patient was transitioned from lenalidomide, bortezomib, and dexamethasone to lenalidomide 10 mg daily as maintenance therapy. She continues to have a complete response to therapy for multiple myeloma and complete hematologic response for her MDS 18 months after treatment initiation. During the course of treatment, she experienced grade 1 fatigue, grade 1 diarrhea, and grade 1 neuropathy. There were no other observed adverse effects.

## Discussion

The patient's case demonstrates successful treatment of concurrently diagnosed multiple myeloma and MDS with isolated del(5q) using lenalidomide, bortezomib, and dexamethasone. The first patient to have both conditions concurrently was reported by Rios et al. in 2000 [4]. This patient had multiple co-morbidities, which prevented her from receiving treatment for her MDS. She passed away shortly after its diagnosis.

We have identified two other case reports describing the simultaneous treatment of multiple myeloma and MDS with isolated del(5q) in the literature [5,6]. In these cases, steroids and varying doses of lenalidomide were tried as treatment options with short-term success. Neither patient received a proteasome inhibitor.

Nolte et al. published a case of multiple myeloma and MDS with isolated del(5q) treated with lenalidomide and dexamethasone [5]. Using lenalidomide at a daily dose of 10 mg with dexamethasone, the patient achieved a complete cytogenetic response of his MDS and good control of his myeloma with an improvement in blood counts. However, he developed secondary acute myeloid leukemia and passed away within four years of treatment initiation.

Ortega et al. described the use of lenalidomide at a daily dose of 10 mg with dexamethasone to achieve a complete hematologic and cytogenetic response of a patient’s MDS with isolated del(5q) [6]. However, only a partial response of the patient’s multiple myeloma was attained. Her lenalidomide dose was eventually increased to 25 mg daily as her myeloma became more active. She passed away from complications of relapsing multiple myeloma within four years of treatment initiation.

In our case, the patient was treated with higher intensity induction therapy for multiple myeloma with an excellent response. We initially feared that the patient’s MDS might unfavorably affect her cell counts when three-drug myeloma therapy was used. She did not have worsening cytopenias during therapy and, in fact, experienced normalization of her blood counts. She continues to do well a year and a half after initiation of treatment.

## Conclusions

In the case presented above, the patient concurrently diagnosed with multiple myeloma and MDS with isolated del(5q) was treated with standard three-drug induction therapy for multiple myeloma. The regimen used was a higher intensity treatment than has been used in this setting. The treatment achieved a stringent complete response of her multiple myeloma and a hematologic and cytogenetic response of her MDS in three months. Use of the regimen was not associated with significant hematologic or other toxicities. Her response was preserved at least 18 months into treatment. Our experience suggests that standard myeloma induction regimens including lenalidomide and a proteasome inhibitor may be considered for treatment of concurrently diagnosed multiple myeloma and MDS with isolated del(5q) and are safe to use in select patients.
